# Risk Perception and Emotion Reaction of Chinese Health Care Workers Varied During COVID-19: A Repeated Cross-Sectional Research

**DOI:** 10.3389/ijph.2021.613057

**Published:** 2021-03-26

**Authors:** Yin Qianlan, Liu Ying, Chen Aibin, Song Xiangrui, Cai Wenpeng, Deng Guanghui, Dong Wei

**Affiliations:** ^1^ Navy Medical University, Shanghai, China; ^2^ The Department of Psychology, Fudan University, Shanghai, China

**Keywords:** protection, negative emotion, risk perception, health care worker, pandemic, COVID-19

## Abstract

**Objectives:** To examine risk perception and negative emotions during two periods of the COVID-19 and provide plausible intervention points for the psychological aid under a stressful condition.

**Methods:** The current study adopted the repeated cross-sectional research and was participated by a cohort of Chinese HCWs who were assigned to work at the current disease resistance line. The between-group information about gender, profession, and location was collected in the demographic questionnaire. Risk perception questionnaire was adapted for COVID-19 to assess risk perception and the Chinese version of emotional self-rating scale (PANAS) was used to evaluate HCWs’ negative emotions.

**Results:** Findings revealed the risk perception and negative emotions of HCWs varied in different gender, profession, location, as well as different periods of COVID-19. Over the different periods, the predominated negative emotion expressed by HCWs varied, but negative emotion was consistently associated with risk perception and could be a significant indicator of risk perception.

**Conclusion:** The significance of this research lies in its examination of risk perception and negative emotions of HCWs confronting the COVID-19 during two periods of the pandemic, which underscored the importance of monitoring the risk perception and negative emotions of HCWs to ensure safety and prevent the return of the pandemics.

## Introduction

On February 12, 2020, the novel coronavirus was officially named COVID-19 by the World Health Organization. Due to its rapid spread, the outbreak of COVID-19 has not only caused widespread public health concern but also caused great psychological stress to the public. Researchers have assessed the mental health burden evoked by COVID-19, showing that Chinese medical personnel bore the brunt of the risk of mental health problems, which affected their attention, cognitive functions, and clinical decisions and even endangered patients due to medical negligence [1]. Additionally, while saving lives, frontline health care workers (HCWs) were also faced with increasing workloads and the risk of infection. According to a report by the National Health Commission of China, 1,716 HCWs had been infected as of February 11, 2020, and 11 of them had died as of February 24, 2020. Hence, HCWs on the front line of the epidemic resistance were facing greater risks than usual, and their perception of these risks could also be an important part of their anti-epidemic work.

Risk perception, a psychological term, refers to an individual’s perception and understanding of various objective risks existing in the outside world and emphasizes the influence of the experience acquired by individuals from intuitive judgment and subjective feelings on cognition [2]. Notably, individuals’ ability to perceive risks associated with the virus is important for rapid changes and adaptation of population behaviors as well as for self-protective measures [3]. Given the importance of human psychological and behavioral factors in staving off pandemics, it is crucial to assess psychological and behavioral responses to the situation and determine how perceived risk is impacted by the pandemic [4]. Moreover, such a public health crisis causing numerous losses in a short period of time harms the psychological well-being of people, which is closely related to how people perceive the danger of a public health crisis [5]. Combined with studies in other fields and relevant literature analysis [6–8], HCWs’ risk perception refers to their knowledge, feelings, and understanding of risk factors and risk characteristics in the health care profession. However, a clear definition is still discussed and explored. Koh et al. carried a study on risk perception of healthcare workers in Singapore during the outbreak of SARS with respect to personal risk, family and social life, stress and workload, and preventive measures [9]. It reported the majority perceived a great risk of personal exposure and feared contracting the disease, and more than half reported increased work stress and workload. A comprehensive study on the influencing factors of risk perceptions has concluded that risk perceptions of HCWs could be clustered into six dimensions: personal safety; physical function; occupational exposures; psychosocial concerns; organization safety; and timing pressure [10]. The key constructs of HCWs’ risk perception included personal health risks, health risks to others, social isolation, and acceptance of risks [11]. A high perception of risk can influence the retention of HCWs within the workforce and their willingness to care for infected patients [12], particularly if they are concerned about their own and their family’s health and safety [13]. In contrast, HCWs whose level of risk perception is very low may be noncompliant with protective behaviors, such as vaccinations and personal protective equipment (PPE), thereby increasing both their own risk as well as increasing the risk of propagation of nosocomial transmission within the hospital and community [14]. As we hypothesized, concerning the particularity of health care professionals and the uncertainty of the disease in patients, HCWs confronting the pandemic have undertaken the risk of occupational exposure combined with a busy working status and shift work, which undoubtedly render their bodies in a state of fatigue and causes them to be busy for long periods of time. Therefore, HCWs’ risk perception would be significantly influenced by the outbreak of COVID-19.

Against the backdrop of epidemics, a paucity of previous studies has assessed the risk perception of HCWs. Koh et al. conducted a qualitative interview schedule and concluded that suffering from infectious diseases significantly impacts nurses’ risk perception [9]. Furthermore, previous studies have shown that the risk perception of a public health crisis is associated with policy support [15]. Theoretically, effective policies can strengthen people’s sense of security, thereby helping to reduce anxiety and restlessness. With a long latency and a rapid spread, there was a lack of policy for meeting emergent needs of the public as well as HCWs, which undoubtedly influenced risk perception in some respects and resulted in negative outcomes of mental health [16]. Moreover, the latest study by Diego et al. showed that HCWs perceived limited access to protective equipment and supports and indicated that organizational safety had impacted the epidemic-related risk for HCWs [17]. However, HCW’s risk perception of COVID-10 was scarcely studied, nor were their related reactions to the perception. Furthermore, many factors play a role in one’s risk perceptions, including those stemming not only from social culture but also from individual differences. Related studies showed that the cognitive and decision-making abilities of HCWs are influenced by their own emotions [18, 19]. This knowledge can inform us of HCWs’ psychological status as reflected by their emotions; as an individual characteristic, such emotion could be associated with perceptions of personal or organizational risks. Previous studies found that HCW’s distress and fears were generated after their exposure to infectious diseases [20, 21]. HCW faced the earliest exposures and then showed some psychological problems, signaling these through negative emotions, such as fear, anxiety, anger, and depression [22, 23]. During the present COVID - 19 epidemic, researchers using an online questionnaire to assess contacted individuals’ depression and anxiety found that these symptoms of depression and anxiety were present in a majority of HCWs, indicating the salient existence of negative emotion [24–26]. A scoping review with 37 studies also showed burnout, stress, and the emotional burden of caring for sick patients were already affecting HCWs before COVID-19 [27]. HCWs’ feelings of fatigue, irritability, frustration and being worn out and the depletion of their supporters’ resources [28] led to fewer positive emotions, poorer performance, and more negative results that would be concerning to patients and society. HCWs with negative emotions are not able to adequately cope with work tasks, thus making it necessary to avoid risks. Therefore, the in-depth understanding of negative emotions of HCWs defending against COVID-19 and their association with risk perception will help to reduce the adverse risk judgments of HCWs due to negative emotions. Importantly, it could also improve HCWs self-protection and quality of clinical treatment.

To explore the potential mechanism of pandemic effects on risk perception of HCW’s after the outbreak of COVID-19, the current study performed repeated cross-sectional research on Chinese HCWs who were assigned to work at the current front lines of disease resistance; the study consisted of assessing the HCWs’ risk perception and evaluating their emotional state. Two cross-sectional assessments were conducted in the same group sample. The first cross-sectional assessment was conducted immediately after the outbreak of COVID-19, when the HCWs were dispatched to the most-hit areas in China; the other occurred upon resumption of normal duties, i.e., the period at which there were no new confirmed cases of COVID-19 and HCWs had returned to their regular positions. Three main hypotheses were proposed:

(1) The HCWs’ perceptions of risk would decrease given their knowledge and the gradual improvements in their protections against the pandemic.
(2) After the peak of COVID-19, HCWs’ overall negative emotions would be eased, and the noteworthy or major subtypes of negative emotions would be different in the two periods.
(3) HCWs’ negative emotions would be significantly related to risk perception in both periods, and there would be differences in the patterns of associations between the subtypes of negative emotions and risk perception across two periods. Literally, negative emotions would play a big part in the explanation of risk perception.

We aimed to explore the association between HCWs’ negative emotions and the level of perceived risk in a public health emergency from the emotional and cognitive behavior perspective, which emphasized the impacts of emotional intervention management on risk perception and self-protection. Being aware the importance of preventing emotionally negative effects may be helpful for designing strategies to improve the mental health of HCWs. In this case, this study would provide plausible intervention points for psychological aid under a stressful condition.

## Methodology

### Participants

The current study was conducted over two time periods over the course of the COVID-19 pandemic. The first period covered the time from the 3rd to 5th of February 2020, when the HCWs had been assigned to medical defense of COVID-19 for 10–15 days while active in the national first-level emergency response. “Period 2” covered the 3rd to 5th of May 2020, when all the dispatched HCWs had returned to their normal positions and resumed their work. Considering the critical period of epidemic prevention and control, face-to-face interviews were impossible for the survey, but an online investigation was available using a web platform. Therefore, we did not have a strict sampling framework; instead, we provided the websites for the targeted groups through WeChat and sorted out the qualified questionnaires based on the criteria for our samples. The study was carried out in a comprehensive hospital in Shanghai with more than 1,000 employed medical staff. However, the HCWs approached were those working with COVID-19 cases, not including those isolated at home; therefore, the number of medical staff on call were only half of the total. This study was performed by the leading physician in charge of health surveillance in the temporary organization against the backdrop of an emergency. The temporary medical team joined a WeChat group including almost 550 members working at the front lines. During the acceleration phase of the epidemic, we invited all the members to complete the online questionnaires through our provided website. Eventually, from the 3rd to 5th of February, 238 participants submitted their questionnaires, for a response rate of 43.3%. However, to avoid random responses and unintentional answers, 220 questionnaires (92.4% valid) were deemed to be qualified under the criteria that the HCWs be on duty during this period and that their time for completing the questionnaires range from 500 ms to1500 ms. The average age of the subjects was 31.91 ± 7.0. Under the same criteria, 304 qualifying questionnaires (95% valid) out of a total of 320 submitted were collected from HCWs during Period 2, with a mean participant age of 30.75 ± 9.28 years; however, 2 questionnaires were excluded for unqualifying response times. There was no age difference between the samples for the two periods within the same group (*t* = 1.56, *p* = 0.120). A detailed description of the between-group differences between the two samples is presented in Table 1. Moreover, because Wuhan was most severely affected and was short of HCWs, many HCWs in our cohort were dispatched from Shanghai to the center of the epidemic. As certified by the leader of the group, no confirmed COVID case was included in our cohort, as the HCWs were working under protection, and only the dispatched personnel had frequent contact with confirmed cases; in contrast, the non-dispatched members, having stayed in a low-risk area, had less contact with confirmed cases (see the supplied material for the calculation information about exposures in our cohort).

**TABLE 1:** Population characteristics stratified for the two study periods (1, during the peak of COVID-19; 2, after the resuming of work).

	Period 1 (n = 220)	Period 2 (n = 304)	*p* value
Mean ages	31.25 ± 7.01	30.42 ± 7.49	0.120^+^
Gender			0.000^×^
Males	38 (17.3%)	18 (5.0%)	
Females	182 (82.7%)	286 (84.1%)	
Profession			0.000^×^
Doctors	61 (27.7%)	19 (6.3%)	
Nurse	159 (72.3%)	285 (93.8%)	
Dispatched to Wuhan			0.002^×^
Not dispatched	151 (68.6%)	245 (80.6%)	
Dispatched	69 (31.4%)	59 (19.4%)	

^+^: *p* value for t test; ^×^: *p* value for chi-square test.

Table 1 Population characteristics stratified for the two study periods (1, during the peak of COVID-19; 2, after the resuming of work).

### Measures

#### Demographic Questionnaire

This questionnaire included information regarding gender, age, and profession. Additionally, considering the effect of exposure level to the virus, we also asked their potential contacts with the confirmed patients and whether they were assigned to Wuhan hospital.

#### Risk Perception Questionnaire

The questionnaire of HCWs’ risk perception for the COVID-19 was referred to the risk perception questionnaire of nursing staff [10]. The questionnaire was self-rated including 14 questions pertaining to six dimensions: personal safety risk (questions 1–2), physical function risk (questions 3–4), occupational exposure risk (questions 5–7), psychosocial evaluation risk (questions 8–10), organizational risk (questions 11–12), and time pressure (questions 13). The rating potentiality of risk was divided into five grades from “never” to “almost always” and was assigned 1-5 points in turn. The higher score for each dimension indicated the higher HCWs’ awareness of the risk. The total score represents the general risk perception. However, in deference to our research design, this questionnaire was adapted. At the beginning of the questionnaire, participants were notified that please recall your experience in the COVID-19 and rate the risks listed below. The self-compiled questionnaire had good internal consistency, and its Cronbach Alpha was 0.905. The dimensional Cronbach’s Alpha was in a range of 0.768–0.854. However, after being shortened, this version no longer attained a good model fit for 6 dimensions but was reliable for one common factor (see the supplied material for confirmatory analysis and factor analysis), which indicated for risk perception after modification (CMIN/DF = 0.911<2; RMSEA<0.05; NFI = 0.998). Hence, we used the weighted sum of dimensional scores for the measured risk perception. An English version of the adapted questionnaire was attached in Supplementary Appendix A.

#### The Self-Assessment Questionnaire for Negative Emotions

The Chinese version of Watson and Tellegen (1988) emotional self-rating scale (PANAS) was adopted in the experiment of an emotional self-rating scale, which was verified by Chinese scholars to be of cross-cultural consistency [29, 30]. The Cronbach’s Alpha of this scale was 0.87. 20 words were contained for describing emotions, including 10 positive words and 10 negative words. The participants were asked to evaluate the emotional intensity they experienced on the current state on a scale of 5, among which 1 meant “very slight or no”; 2 for “a little”; 3 for “moderate”; 4 for “relatively strong”, and 5 for “extremely strong”. As a short survey, seven negative emotions were extracted forming the valid assessment, which included impatience, sadness, upset, tension, guilt, fear, and worry. Considering hostility, irritability, and shame were not common negative emotions for frontline HCWs and were against the professional moral, therefore, they were not considered in the study. Eventually, Cronbach Alpha of the valid short version was above 0.8 in our application.

#### Procedure

Prior to the administration of the questionnaires, the research was evaluated and approved by the ethics committee of the Navy Medical University. Following the requirements of the ethics committee, the written informed consent was obtained from the managers of the hospital, and the consents of the HCWs were from personal e-mails. Data were collected using the online version of the questionnaires. All participants were informed that the researchers were interested in their experiences during COVID-19, the participation was voluntary, and their anonymity was emphasized. HCWs were recruited from the city of Shanghai, China through our corresponding to their hospital managers who helped us to distribute the online questionnaires and emphasize the anonymity of this study. All the qualified questionnaires were scrutinized by our research according to selection criteria-being an HCW and finishing the questionnaires within the standard time.

### Statistical Methods

SPSS24.0 statistical software was used for data processing and analysis. The subtypes of negative emotions were represented by the scores of the sub-dimensions, while the risk perception was the sum of five risk perception dimensions. The Mean and standard deviation (SD) of the statistical score data were reported. The t-test was used to analyze the differences in emotion and risk perception with the control of the between-group factors as these calculated variables were normally distributed in a large sample. In addition, a MANCOVA was performed with seven subtypes of negative emotion (i.e., Impatience, Sadness, Upset, Tension, Guilt, Fear, Caution) to explore the differences in two periods. In this analysis, disparate between-group variables of the two samples were treated as covariates. Then, we computed zero-order correlations between negative emotions and risk perception among the participants for each of the two periods (each period was evaluated separately). Fisher’s test was used to examine the significance of the difference between each two correlation coefficients. Finally, stratified regression analyses were employed to examine the different contribution of negative emotions to indicating levels of risk perception across the two periods with the between-group factors as controlled variables confounding the first strata. Moreover, G*Power 3.1.9.2 was used for calculating the post hoc analysis and all the test levels were at α = 0.05 (all the parameter could be seen in supplied materials). For a two-tailed test at *p* < 0.05, the sample of 220 provided power of 0.95 and 0.99 for effect size of f^2^ = 0.24, and 0.29; the sample of 304 provided power of 0.95 and 0.99 for effect size of f^2^ = 0.21, and 0.24.

## Results

### Between-Group Differences in Negative Emotions and Risk Perception Across the Two Periods

Given that a higher percentage of females than males, a higher percentage of nurses than doctors (this issue was particularly acute for the Period-2 sample) and a small proportion of dispatched HCWs comprised the two samples, therefore, between-group differences in the study variables for each period were examined. Table 2 shows that the gender difference was not significant in the overall negative emotions, except that in Period 2 females showed more worry than males with slight significance (*p* = 0.017, d = 0.40). Notably, the professional difference in the two periods was not consistent. Only in Period 1, doctors rated higher than nurses in the general risk perception (*p* = 0.027, d = 0.34) and negative emotions including impatience (*p* < 0.001, d = 0.51), sadness (*p* = 0.002, d = 0.52), upset (*p* = 0.01, d = 0.38), and tension (*p* = 0.012, d = 0.37). In Period 2, no significant difference of risk perception was discovered in HCWs’ location (*p* = 0.209, d = 0.07), however, compared to dispatched HCWs, those not dispatched to Wuhan presented more negative emotions including impatience (*p* = 0.018, d = 0.32), sadness (*p* = 0.016, d = 0.30), tension (*p* = 0.003, d = 0.39), fear (*p* = 0.038, d = 0.25) and worry (t = 2.57, *p* = 0.011, d = 0.32). As for the between-group differences confounded with the studied variables, in subsequent analyses, these differences were treated as covariates.

**TABLE 2:** Negative emotions and risk perception during period 1 (during the peak of COVID-19) and 2 (after the resuming of work) stratified for gender, occupation and dispatch to Wuhan.

	Gender (Mean ± SD)				Profession (Mean ± SD)				Dispatch (Mean ± SD)			
**Period 1**	**Males**	**Females**	**t**	**P**	**Doctors**	**Nurses**	**t**	**p**	**Shanghai**	**Wuhan**	**t**	**p**
Risk perception	38.95 ± 9.71	37.36 ± 9.48	0,94	0.350	39.92 ± 9.45	36.75 ± 9.42	2.23	0.027	37.43 ± 9.71	38.07 ± 9.13	−0.463	0.643
Impatience	1.79 ± 0.91	1.53 ± 0.83	1.71	0.088	1.90 ± 1.00	1.45 ± 0.74	3.63	0.000	1.60 ± 0.83	1.52 ± 0.87	0.66	0.510
Sadness	1.61 ± 0.86	1.49 ± 0.82	0.75	0.453	1.84 ± 1.00	1.39 ± 0.72	3.22	0.002	1.52 ± 0.86	1.49 ± 0.76	0.25	0.800
Upset	1.92 ± 0.82	1.71 ± 0.93	1.31	0.192	2.00 ± 1.00	1.65 ± 0.86	2.60	0.010	1.79 ± 0.95	1.64 ± 0.80	1.19	0.236
Tension	2.13 ± 1.00	1.87 ± 0.88	1.62	0.107	2.16 ± 1.00	1.82 ± 0.85	2.55	0.012	1.88 ± 0.96	2.00 ± 0.75	−1.00	0.318
Guilt	1.26 ± 0.50	1.32 ± 0.66	−0.49	0.626	1.34 ± 0.60	1.30 ± 0.65	0.51	0.613	1.28 ± 0.64	1.36 ± 0.64	−0.84	0.404
Fear	1.47 ± 0.80	1.164 ± 0.85	−1.13	0.259	1.74 ± 0.91	1.57 ± 0.80	1.36	0.175	1.59 ± 0.87	1.67 ± 0.76	−0.63	0.527
Worry	1.45 ± 0.72	1.51 ± 0.85	−0.43	0.666	1.66 ± 0.89	1.44 ± 0.79	1.74	0.083	1.54 ± 0.90	1.42 ± 0.65	1.09	0.279
												
Period 2										(Back to Shanghai)		
Risk perception	32.72 ± 7.03	34.64 ± 9.92	0.42	0.421	32.58 ± 7.97	34.65 ± 9.88	−0.90	0.371	34.87 ± 10.07	33.08 ± 8.36	1.26	0.209
Impatience	1.5 ± 0.92	1.66 ± 0.86	−0.75	0.452	1.47 ± 0.84	1.66 ± 0.86	−0.91	0.361	1.7 ± 0.89	1.44 ± 0.70	2.39	0.018
Sadness	1.39 ± 0.70	1.41 ± 0.73	−0.09	0.925	1.32 ± 0.58	1.41 ± 0.74	−0.55	0.584	1.44 ± 0.76	1.24 ± 0.54	2.44	0.016
Upset	1.83 ± 1.10	1.8 ± 0.92	0.13	0.897	1.68 ± 1.00	1.81 ± 0.92	−0.59	0.555	1.85 ± 0.94	1.63 ± 0.83	1.66	0.098
Tension	1.33 ± 0.60	1.52 ± 0.82	−0.97	0.331	1.26 ± 0.56	1.53 ± 0.82	−1.94	0.065	1.57 ± 0.85	1.29 ± 0.56	3.08	0.003
Guilt	1.11 ± 0.32	1.24 ± 0.57	−0.94	0.350	1.11 ± 0.32	1.24 ± 0.57	−1.67	0.106	1.23 ± 0.53	1.24 ± 0.68	−0.11	0.914
Fear	1.22 ± 0.43	1.29 ± 0.64	−0.45	0.655	1.26 ± 0.56	1.29 ± 0.63	−0.17	0.868	1.31 ± 0.66	1.17 ± 0.42	2.09	0.038
Worry	1.11 ± 0.32	1.33 ± 0.70	−2.55	0.017	1.21 ± 0.54	1.33 ± 0.70	−0.71	0.479	1.36 ± 0.74	1.17 ± 0.42	2.57	0.011

Table 2. Negative emotions and risk perception during period 1 (during the peak of COVID-19) and 2 (after the resuming of work) stratified for gender, occupation and dispatch to Wuhan.

### Differences in Negative Emotions and Risk Perception Across the Two Periods

To test our first and second hypotheses, a multivariate analysis was used to reveal the main effect of the period and prove it to be significant [Wilks’λ = 0.874, F (8, 513) = 9.21, *p* < 0.01, ηρ^2^ = 0.13]. The univariate main effects were examined and are reported in Table 3. Gender [F (8, 513) = 2.71, *p* = 0.006, ηρ^2^ = 0.04] , the professional difference [F (8, 513) = 3.45, *p* = 0.001, ηρ^2^ = 0.05] and whether being dispatched or not [F (8, 513) = 2.13, *p* = 0.032, ηρ^2^ = 0.03] were significant covariates. After the effect of the three variables controlled, the period had a main effect on tension, fear, worry, and risk perception (Table 3). The negative emotions, including tension, fear, and worry, and risk perception were all significantly higher during Period 1 than they were during Periods 2 (mean differencetension = −0.38, *p* < 0.001, mean differencefear = −0.30, *p* < 0.001; mean differenceworry = −0.16, *p* = 0.024; mean differencerisk perception = −2.76, *p* = 0.002). No significant differences were found between the levels of impatience, sadness, upset, and guilt during the two periods.

**TABLE 3:** Negative emotions and risk perception stratified for 1 (during the peak of COVID-19) and 2 (after the resuming of work).

	Period 1 (Mean ± SD)	Period 2 (Mean ± SD)	F	p	ηρ^2^
Risk perception	37.63 ± 9.51	34.83 ± 9.78	9.446	0.002	0.018
Impatience	1.58 ± 0.84	1.65 ± 0.86	2.281	0.132	0.004
Sadness	1.51 ± 0.83	1.40 ± 0.73	0.493	0.483	0.001
Upset	1.75 ± 0.91	1.81 ± 0.93	1.173	0.279	0.002
Tension	1.92 ± 0.90	1.51 ± 0.81	22.739	0.000	0.042
Guilt	1.31 ± 0.64	1.23 ± 0.56	1.740	0.188	0.003
Fear	1.61 ± 0.84	1.29 ± 0.62	19.596	0.000	0.036
Worry	1.50 ± 0.83	1.32 ± 0.70	5.117	0.024	0.010

In the Multivariate Analysis of Variance, gender, occupation and dispatch were controlled as concomitant variable; ηρ^2^ (partial eta squared) is the variance explained by a given variable of the variance remaining after excluding variance explained by other predictors.

Table 3 Negative emotions and risk perception stratified for period 1 (during the peak of COVID-19) and 2 (after the resuming of work).

### The Association of Negative Emotions to the Indication of Stress Reactions Across the Two Periods

The correlation between all types of negative emotions and risk perception were all significant (all the *p* values were less than 0.01) and the strength of their associations remained the same across two periods (|z|<1.96), which was shown in Supplementary Appendix B. To closely examine the complex associations between negative emotions and risk perception among HCWs, the regression analyses were employed for each period with the general risk perception served as the outcome. In strata 1, the indicators were dummy-coded variables including gender (0 = female, 1 = male), profession (0 = doctor, 1 = nurse), and location (0 = Shanghai, 1 = Wuhan). In strata 2, the main effects of different types of negative emotions were examined (Table 4). The results indicated that under the control of the covariates of between-group difference, in period 1, the negative emotion of worry could contribute most to risk perception (B = 2.67, *p* = 0.005, SE = 0.95), while in period 2, the negative emotion of tension turned to be the significant contributor (B = 2.95, *p* = 0.006, SE = 1.06).

**TABLE 4:** Multivariable associations between gender, profession, location, negative emotions (independent variables) and risk perceptions (dependent variables), in 1 (during the peak of COVID-19) and 2 (after the resuming of work).

		R^2^	Period 1 (N = 220)				R^2^	Period 2 (N = 304)			
			B	SE	Beta	P		B	SE	Beta	P
Strata 1		0.026					0.006				
	Gender (1 = male)		2.15	2.40	0.09	0.370		0.29	3.58	0.01	0.935
	Occupation (1 = nurse)		‒4.43	2.03	‒0.21	0.031		0.91	3.52	0.02	0.796
	Location (1 = Wuhan)		0.28	1.38	0.01	0.839		‒1.50	1.56	‒0.06	0.334
Strata 2		0.274					0.273				
	Gender (1 = male)		‒0.38	2.20	‒0.02	0.862		1.62	3.13	0.04	0.604
	Occupation (1 = nurse)		‒1.01	1.89	‒0.05	0.595		‒0.08	3.07	0.00	0.981
	Location (1 = Wuhan)		0.86	1.25	0.04	0.49		0.38	1.38	0.02	0.786
	Impatience		0.19	1.16	0.02	0.869		0.73	0.88	0.06	0.407
	Sadness		0.03	1.08	0.01	0.979		2.14	1.12	0.16	0.057
	Upset		1.45	1.10	0.14	0.188		1.43	0.96	0.14	0.139
	Tension		1.25	0.99	0.12	0.205		2.95	1.06	0.24	0.006
	Guilt		‒0.61	1.09	‒0.04	0.575		‒0.64	1.24	‒0.04	0.606
	Fear		1.46	1.20	0.13	0.226		0.56	1.50	0.04	0.708
	Worry		2.67	0.95	0.23	0.005		‒0.22	1.23	‒0.02	0.856

** *p* < 0.01; Risk perception entered as the dependent variable.

Table 4. Multivariate associations between gender, profession, location, negative emotions (independent variables) and risk perceptions (dependent variables), in 1 (during the peak of COVID-19) and 2 (after the resuming of work).

## Discussion

Our findings revealed that the risk perception and negative emotions of HCWs varied with regard to gender, profession, and location, as well as between the different periods of COVID-19. Our findings also revealed differences in the association between risk perception and negative emotions across the two periods. The levels of tension, fear, worry, and risk perception were higher during Period 1 than they were during Period 2 of COVID-19. Between the different periods, worry was found to be closely related to and a significant indicator of high-level risk perception in Period 1; in contrast, the indicative type of negative emotion turned to be tension in Period 2.

The current study examined differences in risk perception and negative emotions and the varied associations between those variables among HCWs across two periods of COVID-19. The first hypothesis, that risk perception and negative emotions would decrease across two successive periods of COVID-19, was supported by our findings, which was consistent with the development of the pandemic. Our study was consistent with longitudinal and cohort studies conducted after the outbreak of SARS. These studies found that during the initial phases of the SARS outbreak, risk perception steadily increased and then leveled off in later phases [21, 31]. As explained by other studies of public risk perception, the risk associated with COVID-19 was novel and poorly known, disrupting the safety system of people’s cognitions, which was reflected by an explosion in information seeking [32]. However, as an infectious disease, the increase in risk perception could be neutralized by effective vaccines or behavioral containment measures, which would likely lead to less concern regarding the notion of risk. As regards the negative emotion components, significant decreases were found in tension, fear, and worry across the two periods studied. In Period 1, during the peak of the pandemic, initial knowledge about the origin and life course of the newly emerging virus was lacking, and visible effects were delayed; therefore, unknown risks and threats to public health resulted [11]. Another study also proved that due to increases in the uncertainty of risk, risk perception grew and became more linked to emotional appraisals [33]. HCWs, as the first-line warriors for public health, were dispatched to the hardest-hit areas or were mandated to work under the threat of infection, which could arouse negative emotions in them, such as tension, fear, and worry. Conversely, in Period 2, when the pandemic was contained through protective measures, the threat to HCWs had been handled. Meanwhile, tension, fear, and worry were found to have gradually been relieved, and the risk perception of the pandemic was reduced. However, the risk was not nullified, as nonspecific treatment and a vaccine were being developed at that time; accordingly, negative emotions did not wear off. For instance, our study showed the HCWs’ emotion of upset increased in the later period, which was consistent with a study of the German public study showing that one month after the outbreak, the risk perception of getting infected with COVID-19 had persisted steadily in general population [34]. Moreover, it is suggested that fears regarding the COVID-19 pandemic could be a long-lasting consequence rather than as pathologic reaction [35], and data prove that anxiety regarding the COVID-19 pandemic leads to significant burdens in daily life [34, 36]. From a practical view of our results, in the acute stage of COVID-19, tension, fear, and worry are likely to be imminent and therefore hamper the rational perception about gains and losses related to protective behaviors of HCWs, not only for themselves but also for their patients. In such cases, the proper coping mechanisms for these emotions would be used to fend off invisible losses and long-term consequences. In the later stage, risks were downplayed due to the emotional coping strategy, which would have redoubled the effects of the protective behaviors. Furthermore, the acceptance of anxiety and negative emotions and seeking support or knowledge decreased the negative effects of COVID-19. To ensure safety, it would be better to monitor the risk perception and negative emotions of HCWs for the sake of preventing the return of the pandemics.

Notably, some between-group differences were detected in the analysis. In China’s COVID-19 response, female nurses and community health workers were the first line of defense against the outbreak, which could explain why the two samples were dominated by female HCWs, most of whom were nurses. Therefore, the study might be less convincing with regard to its interpretation of gender differences. However, as shown in related studies, female HCWs have exhibited greater vulnerability to distress and depression [37, 38]. Particularly, doctors showed more negative feelings, such as impatience, sadness, upset, and tension in Period 1. These findings reflected the hardship borne by doctors as they experienced overwhelming stress from saving the lives of threatened patients and faced numerous patients waiting for care. Meanwhile, risk perception was also higher in doctors than nurses. It can be supposed that frontline nurses could have better training to deal with crisis, which is in agreement with studies that have found experienced HCWs to be less affected by stress [39]. Conversely, in Period 2, nurses showed higher risk perception than doctors due to nurses in this stage having to provide greater support for and closer contact with the patients. In fact, various explanations can be posited for the many comparisons that have been made between HCWs and other groups. For example, a large-scale study of Chinese HCWs showed that risk factors for anxiety and depression included being employed as a nurse and being a frontline HCW, [40], while it was also found that HCWs experienced anxiety about their own and their family’s safety but maintained the professional obligation to effectively complete their work [24]. Frontline HCWs have exhibited significantly lower levels of burnout and less worry about becoming ill, as they perceive themselves as having more control over their situation and may appreciate a closer proximity to authority; however, increased stress related to the unexpected development of the pandemic and longer work hours might have burned HCWs out [41], which cooccurred with negative emotions deteriorating their perception of the situation [42, 43]. Moreover, in the late stage of COVID-19, undispatched HCWs scored higher on the subscales of impatience, sadness, tension, and worry compared to HCWs dispatched to Wuhan, despite their risk perception not being significantly different. This finding could partly be explained by dispatched HCWs having received more psychological and substantial help, as many concern were focused on the epidemic’s center. This results was also consistent with a study by Cao et al., who found that levels of burnout and emotional distress were not highly elevated within their sample; instead, connection with family members via technology or the telephone was the most prevalent coping mechanism [44]. Therefore, healthy coping strategies, including team or organizational encouragement and rational thinking are important because the offer a chance for reflection and for developing a sense of professional responsibility, resulting in growth. Nevertheless, more studies should focus on the difference between dispatched HCWs and those who were undispatched to clarify the pandemic’s impacts on HCWs’ mental health, considering that many potential factors (e.g., including the protective equipment, work experiences) remain intertwined with each other and may affect the explanations. In our cohort, all the HCWs were geared with protections, and the sources for medical supplies were ensured as the Chinese government took an urgent mobilization; therefore, issues for protection equipment were possibly not perceived to be as worrying or frustrating as other counties where the need for personal protective equipment exceeded the supplied [45]. Moreover, a lot of Chinese HCWs petitioned to be dispatched to the hardest hit area and most of them were rich in the medical practices or related experiences. This group of HCWs set a model for the rest of coworkers, encouraging the youngster and forming a inspiring organizational atmosphere. These potential factors could partly explain why dispatched HCWs showed slight negative emotions. Hence, future discoveries for the potential factors could share more lessons for defending the pandemic and protecting our front-line HCWs.

After controlling for the confounders of gender, profession, and location, further exploration for the association between negative emotions and risk perception not only provided supports for our third hypothesis but also for the existence of emotion-based risk perception. It has been suggested that automated incentive or alarm signals linked to pleasant or unpleasant “gut feelings” often precede cognitive reasoning [46]. In a similar vein, processing theories have emphasized the importance of an emotion–cognition pathway [47, 48]. Negative emotions serve a dual role: as information and as motivation. In an acute-threat situation such as COVID-19, emotion might gain more immediate importance with regard to their informational role, as people especially use negative feelings associated with a target to evaluate that target negatively. Under these circumstances, when experts are unable to make tentative statements and provide partly contradictory prognoses and recommendations, cognitive risk assessments might be severely hampered by a lack of evidence-based information. Consequently, individuals might have little choice but to rely on experiential judgment largely affected by emotional state. Hence, the uncertainty of COVID-19 aroused HCWs’ negative emotions, which resulted in the negativity of perceived risk. Our study has shed light on the effects of emotion on risk perception through the use of a repeated cross-sectional method. As the data analysis showed, worry could have been a prominent contributor to the higher level of risk perception evident immediately after the outbreak of the pandemic. However, the prominent components of negative emotions may have varied over the course of a pandemic; thus, in our second studied COVID-19 period, tension, reflecting the local situation and the severity of the pandemic, played a key role in risk perception. However, what role exactly an emotion plays in interaction with risk-related cognition and behaviors is still an issue in need of clarification. Only a minority of the subgroup of studies having investigated the possible indicative role of risk perception for protective behaviors have actually considered emotion-based explanations [49–51]. The motivational role of negative emotions is controversial due to the lack of opportunity for studying the complexity of the decision-making process, which was also bound to affect the extent to which findings for the relationship between risk perception and protective behaviors can be interpreted. Thus, a more systematic application of multifactor models, including emotion-based models, would allow for far more complex insights into the workings of risk perceptions in shaping behavior.

### Implications

Our HCWs are bravely living in a constant state of psychological stress. Perceived risk, lack of control, high levels of stress, and negative emotional arousal are risk factors influencing HCWs’ well-being. The long-term effects of stress can also result in posttraumatic stress disorder, anxiety and depression. Potential consequences of unmanaged stressful conditions will have implications not only for HCWs but also for patients and health systems: physical problems, irrational decision-making, diminished quality of care, absenteeism, and negative attitude. Emphatically, negative emotions affected HCWs’ risk perceptions greatly related to protective behaviors. HCWs as a mediating channel between health agencies and the public play a major role in the attitudes regarding compliance of the general public with policies or vaccination. However, they also based their attitude more on emotions and perceived the risk tangible and relevant. It was reported even the health care professionals are often in a situation of cognitive dissonance due to emotions negating their perceptions [52]. Thus, it is imperative to implement actions and interventions to care for HCWs during and after emergencies to prevent a more costly situation. Moreover, emotion coping strategies should be valued and trained by HCWs as they would mix emotions with analytic analysis and create a complex risk perception with a negative bias. Planning and building the risk communication plan while address HCWs’ negative emotions, especially for worries, should be implemented immediately after the outbreak of the crisis, and sequential proactive measures should be targeted for ameliorating the emotional tension for HCWs.

### Limitations

The current study has some limitations that should be addressed. First, the data were collected via phones during COVID-19. Therefore, some degree of potential sample bias should be considered. On the one hand, there was a low responding rate from front-line HCWs, as they were overwhelmed by works in both periods and missed the invitation, or their phones were tightly managed in case of potential transmission. Inevitably, the responding rate of our investigation was lower than expected. On the other hand, the two samples each included more percentages of females vs. males and nurses vs. doctors. This uneven percentage was particularly salient for the Period 2 sample. Therefore, future studies should recruit a larger sample. Moreover, many potential factors were neglected in our research, such as variations in age and prior experience within our samples, due to the limited time for the investigation; these oversights indicate the need for great caution in drawing extensive implications from the findings. Another caveat is that the current study employed a cross-sectional repeated design to examine differences in the levels of negative emotions and risk perception. Hence, even if between-group characteristics were relatively stable and controlled for, the samples at Period 1 and Period 2 were “the same” only in regard to the factors presumed to be relevant when making comparisons with outcome measures. Because we did not engage in a strict sampling regimen, there was no way to perform the analysis only on people having participated at both time points. It is true that we performed our analysis with two respective subgroups, instead of one pooled group; nevertheless, if units within time points shared unmeasured commonalities that were not fully considered in our study, such as age and education, then the standard errors may be incorrect. Furthermore, if one filters the time component via fixed effects to control for between-time-point effects, it limits the exploration to static processes. Pooling has its problems; therefore, a more extensive study could help to critique the validity of the time-point comparison. Moreover, as a snapshot of the emotional and cognitional responses of HCWs during the two periods of COVID-19, some interaction between multiple factors and their multidimensional causality could not be accurately explained. For example, the follow-up research could achieve more convincing results about the effect of being forcefully dispatched to high-risk areas or desperately short on personal protective equipment, which could contribute to the identifying targeted help services for HCWs.

### Conclusion

The significance of this research lies in its examination of risk perception and negative emotions of HCWs confronting the COVID-19 during two periods of the pandemic. The findings showed both risk perception and negative emotions of HCWs were affected by the COVID-19. Additionally, the predominated negative emotions of HCWs presented varied with time course, but the relations to risk perception were constant and could be a significant indicator of risk perception. These findings underscore the importance of negative emotions as a significant factor for risk perception of HCWs enduring the challenge of the pandemic within the Chinese population. More importantly, our results underscore studying the change of pandemic risk perceptions and their “true” influence over time remains an important objective, requiring not only studies starting after potential future outbreaks but also long-term surveillance studies which are initiated before an actual outbreak occurs.

## Data Availability

The raw data supporting the conclusions of this article will be made available by the authors, without undue reservation.

## Abbreviations

COVID-19, Coronavirus disease 2019; HCWs, Health care workers; PANAS, Positive and negative emotions scale; PPE, Personal protective equipment; SARS, Severe Acute Respiratory Syndromes; MANOVA, Multivariate analysis of variance; SD, Standard deviations.
