# Supplementary material for: Risk Perception and Emotion Reaction of Chinese Health Care Workers Varied During COVID-19: A Repeated Cross-Sectional Research
Source: Int J Public Health. 2021 Mar 26;66:613057. doi: 10.3389/ijph.2021.613057 (PMC8565291; doi:10.3389/ijph.2021.613057)
Supplement: Supplementary file 1 [file DataSheet1.PDF]

The calculated calculation information for about exposures of in our cohort

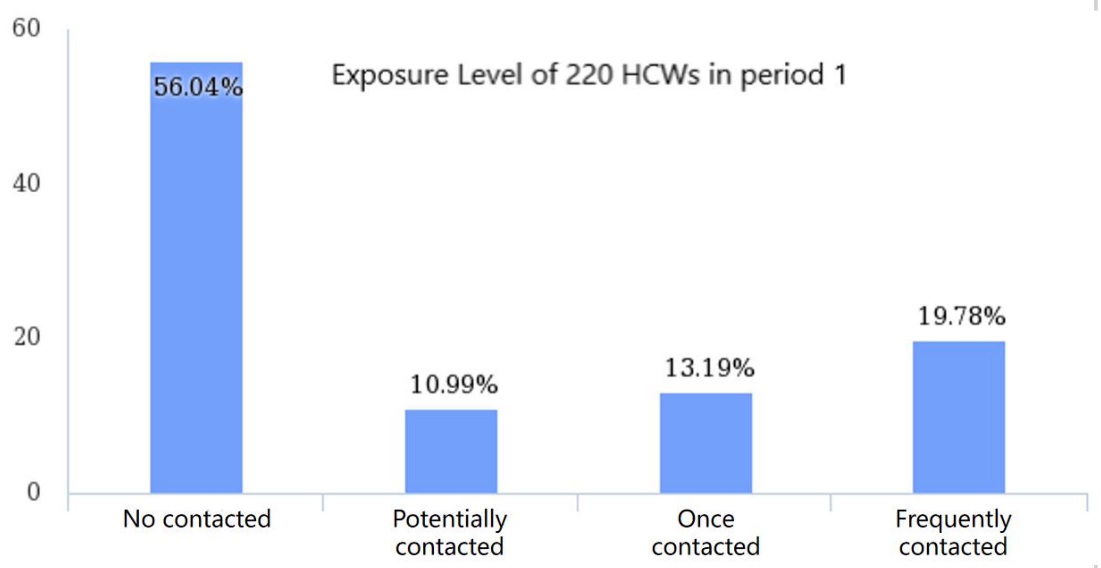

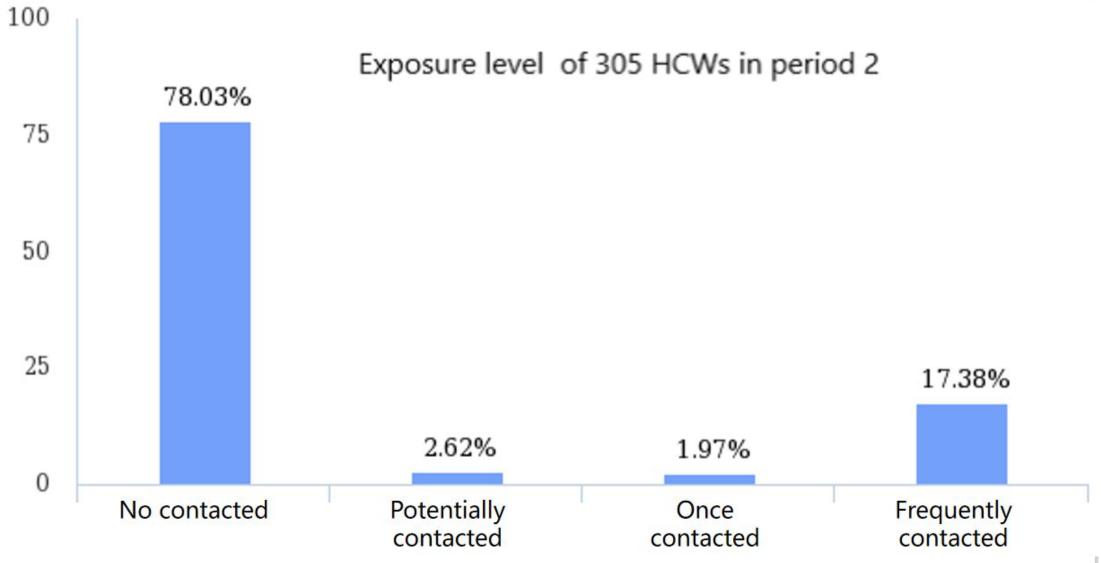

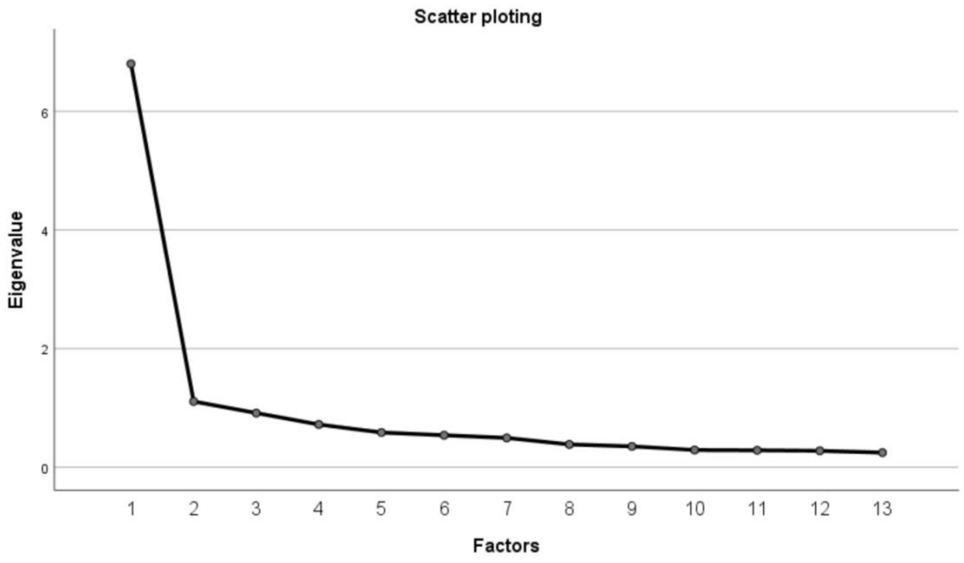

Total Variance Explained

Component	Initial Eigenvalues			Extraction Sums of Squared Loadings			Rotation Sums of Squared Loadings		
	Total	% of Variance	Cumulative %	Total	% of Variance	Cumulative %	Total	% of Variance	Cumulative %
1	6.800	52.311	52.311	6.800	52.311	52.311	4.083	31.404	31.404
2	1.109	8.533	60.844	1.109	8.533	60.844	3.827	29.440	60.844
3	.913	7.024	67.868						
4	.721	5.546	73.414						
5	.586	4.505	77.919						
6	.540	4.155	82.074						
7	.495	3.804	85.878						
8	.384	2.957	88.835						
9	.352	2.709	91.544						
10	.291	2.237	93.781						
11	.285	2.193	95.974						
12	.277	2.130	98.104						
13	.246	1.896	100.000						

Extraction Method: Principal Component Analysis.

Communalities

	Initial	Extraction
1.你常担心医治过程中会出现纠纷	1.000	.637
2.你常担心失控患者会对自己造成人身伤害	1.000	.724
3.你常担心高压力状态会影响自己的身体健康	1.000	.632
4.你常担心因为接触传染患者会感染自己	1.000	.692
5.你常担心自己被感染后被治愈的可能会很小	1.000	.590
6.你常担心自己会被人隔离	1.000	.441
7.你常担心操作过程中被污染的锐器割伤或针具刺伤	1.000	.588
8.你常担心不能及时发现患者病情变化而延误抢救	1.000	.617
9.你常担心同事或病人对自己的负面评价	1.000	.549
10.你常担心自己工作时会出现差错	1.000	.718
11.你常担心医院医疗资源不够，医院防护和隔离措施保障不足	1.000	.550
12.你常担心疫情状况会进一步恶化	1.000	.637
13.你常担心自己陪家人的时间可能会越来越少	1.000	.535

Extraction Method: Principal Component Analysis.

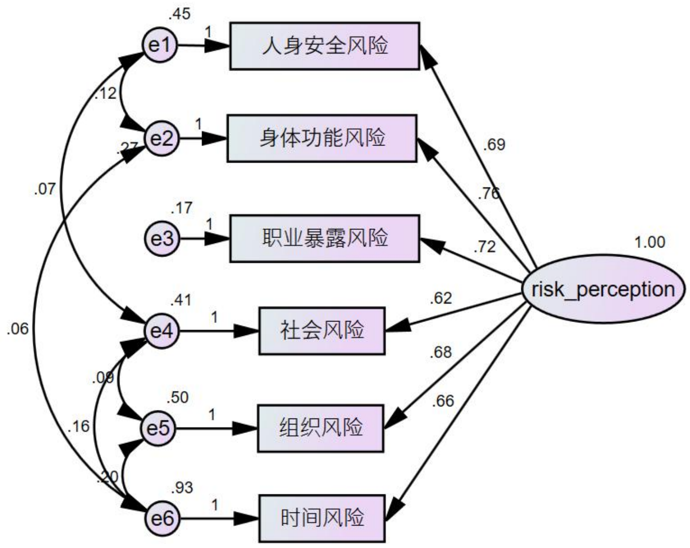

Regression Weights: (Group number 1 - Default model)

			Estimate	S.E.	C.R.	P	Label
身体功能风险	<---	risk_perception	.762	.035	21.541	***	
职业暴露风险	<---	risk_perception	.720	.031	23.018	***	
社会风险	<---	risk_perception	.623	.036	17.078	***	
组织风险	<---	risk_perception	.675	.040	16.937	***	
时间风险	<---	risk_perception	.664	.051	12.923	***	
人身安全风险	<---	risk_perception	.694	.040	17.487	***	

Covariances: (Group number 1 - Default model)

			Estimate	S.E.	C.R.	P	Label
e5	<-->	e6	.204	.036	5.726	***	
e1	<-->	e2	.125	.023	5.307	***	
e4	<-->	e6	.155	.031	5.018	***	
e1	<-->	e4	.074	.019	3.868	***	
e4	<-->	e5	.094	.024	3.901	***	
e2	<-->	e6	.058	.023	2.526	.012	

Variances: (Group number 1 - Default model)

	Estimate	S.E.	C.R.	P	Label
risk_perception	1.000				
e1	.449	.035	12.829	***	
e2	.269	.026	10.232	***	
e3	.171	.021	8.223	***	
e4	.410	.030	13.631	***	
e5	.497	.036	13.692	***	
e6	.928	.063	14.631	***	

Model Fit Summary

CMIN

Model	NPAR	CMIN	DF	P	CMIN/DF
Default model	18	2.734	3	.434	.911
Saturated model	21	.000	0		
Independence model	6	1721.654	15	.000	114.777

RMR, GFI

Model	RMR	GFI	AGFI	PGFI
Default model	.005	.998	.988	.143
Saturated model	.000	1.000		
Independence model	.445	.372	.120	.265

Baseline Comparisons

Model	NFI Delta1	RFI rho1	IFI Delta2	TLI rho2	CFI
Default model	.998	.992	1.000	1.001	1.000
Saturated model	1.000		1.000		1.000
Independence model	.000	.000	.000	.000	.000

RMSEA

Model	RMSEA	LO 90	HI 90	PCLOSE
Default model	.000	.000	.071	.819
Independence model	.466	.448	.485	.000

CMIN/DF
CMIN/DF is the minimum discrepancy, , (see Appendix B) divided by its degrees of freedom:
"... it seems clear that a ratio > 2.00 represents an inadequate fit." (Byrne, 1989, p. 55).Byrne, B.M. (1989). A primer of LISREL: Basic applications and programming for confirmatory factor analytic models. New York: Springer-Verlag.
GFI is less than or equal to 1. A value of 1 indicates a perfect fit.
RMSEA incorporates no penalty for model complexity and will tend to favor models with many parameters.
"Practical experience has made us feel that a value of the RMSEA of about .05 or less would indicate a close fit of the model in relation to the degrees of freedom. This figure is based on subjective judgment. It cannot be regarded as infallible or correct, but it is more reasonable than the requirement of exact fit with the RMSEA = 0.0. We are also of the opinion that a value of about 0.08 or less for the RMSEA would indicate a reasonable error of approximation and would not want to employ a model with a RMSEA greater than 0.1." (Browne and Cudeck, 1993)
Gpower results
1.t-test for the effect size(d) between 38 males and 182 females in period 1

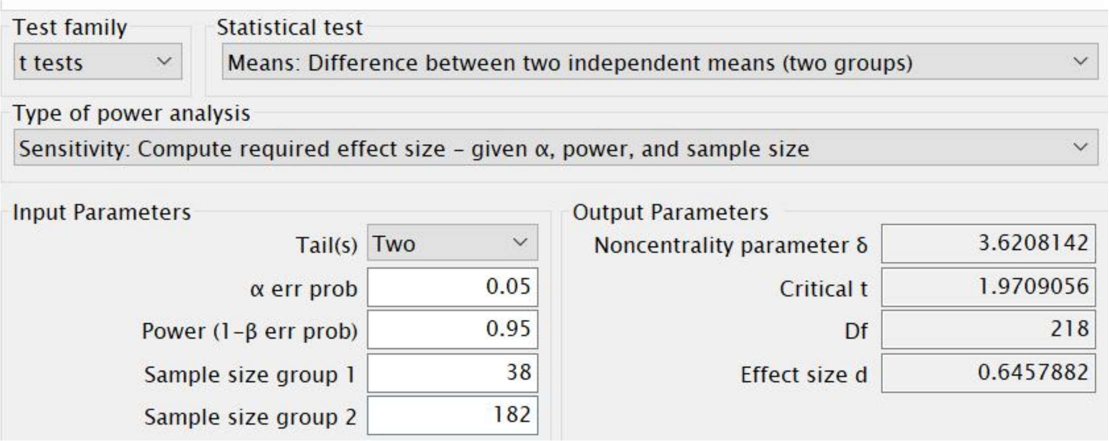

2. t-test for the effect size(d) between 61 doctors and 159 nurses females in period 1

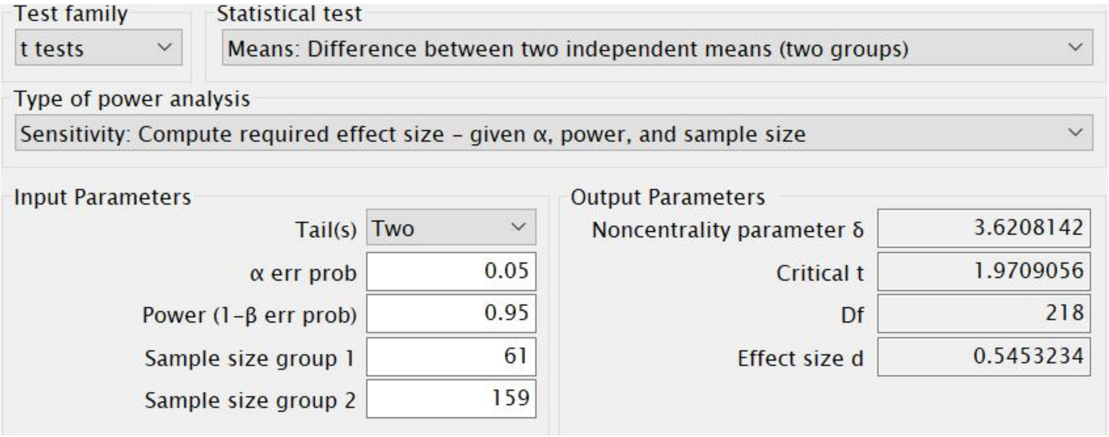

3. t-test for the effect size(d) between 69 dispatched HCWs and 151 not dispatched HCWs in period 1

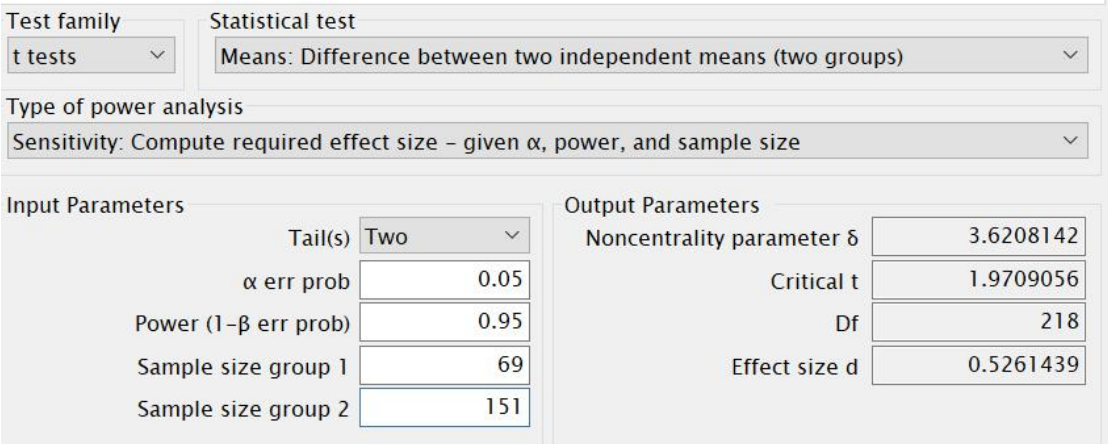

4. t-test for the effect size(d) in period 2

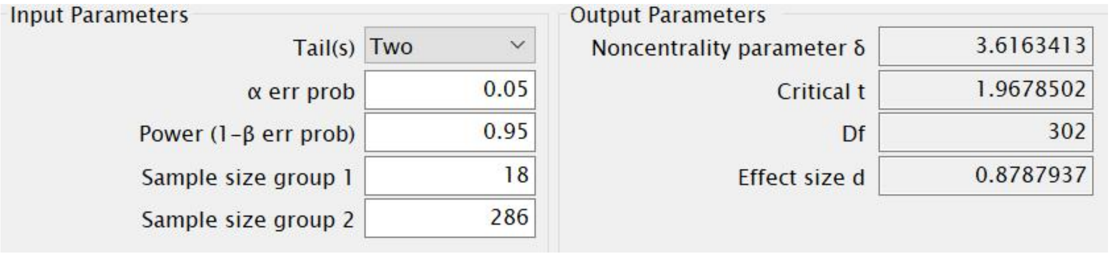

Input Parameters		Output Parameters	
Tail(s)	Two	Noncentrality parameter δ	3.6163413
α err prob	0.05	Critical t	1.9678502
Power (1−β err prob)	0.95	Df	302
Sample size group 1	19	Effect size d	0.8568543
Sample size group 2	285		

Input Parameters		Output Parameters	
Tail(s)	Two	Noncentrality parameter δ	3.6163413
α err prob	0.05	Critical t	1.9678502
Power (1−β err prob)	0.95	Df	302
Sample size group 1	59	Effect size d	0.5244416
Sample size group 2	245		

Central and noncentral distributions | Protocol of power analyses

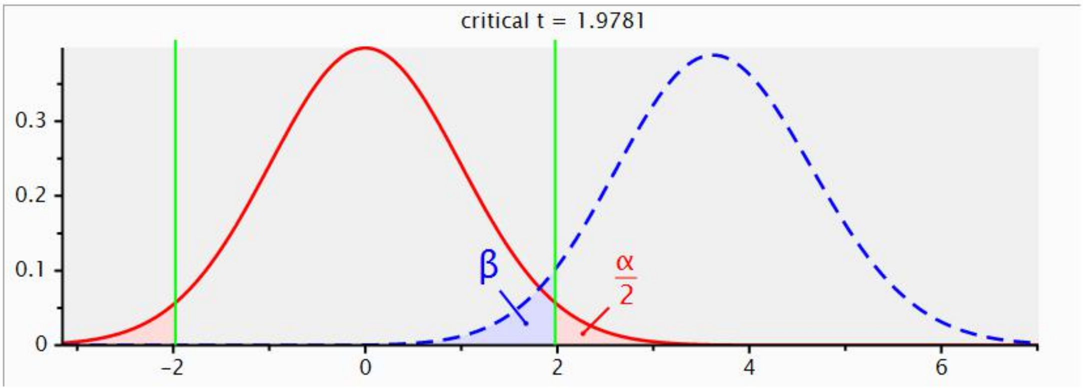

Test family: t tests

Statistical test: Correlation: Point biserial model

Type of power analysis: A priori: Compute required sample size – given α, power, and effect size

Input Parameters		Output Parameters	
Tail(s)	Two	Noncentrality parameter δ	3.6404323
Determine => Effect size |ρ|	0.3	Critical t	1.9780988
α err prob	0.05	Df	132
Power (1−β err prob)	0.95	Total sample size	134
		Actual power	0.9509217

Input Parameters		Output Parameters	
Tail(s)	Two	Noncentrality parameter δ	4.6645731
Determine => Effect size |ρ|	0.3	Critical t	1.9709056
α err prob	0.05	Df	218
Total sample size	220	Power (1−β err prob)	0.9963623

Input Parameters			Output Parameters	
	Tail(s)	Two	Noncentrality parameter δ	5.4832412
Determine =>	Effect size |ρ|	0.3	Critical t	1.9678502
	α err prob	0.05	Df	302
	Total sample size	304	Power (1-β err prob)	0.9997724

系数[a]

模型		未标准化系数 B	未标准化系数 标准错误	标准化系数 Beta	t	显著性	B 的 95.0% 置信区间 下限	B 的 95.0% 置信区间 上限
1	(常量)	33.401	5.335		6.261	.000	22.902	43.899
	您的性别	.290	3.578	.007	.081	.935	-6.751	7.331
	护理分类	.909	3.517	.023	.258	.796	-6.013	7.831
	您现在所在省份	-1.504	1.555	-.061	-.967	.334	-4.565	1.557
2	(常量)	20.477	4.817		4.251	.000	10.997	29.956
	您的性别	1.622	3.125	.039	.519	.604	-4.529	7.773
	护理分类	-.075	3.067	-.002	-.024	.981	-6.110	5.961
	您现在所在省份	.375	1.375	.015	.272	.786	-2.332	3.081
	请对您当前的情绪状态进行评估—急躁	.728	.876	.064	.831	.407	-.996	2.452
	哀伤	2.138	1.120	.160	1.910	.057	-.065	4.341
	心烦	1.428	.963	.135	1.483	.139	-.468	3.324
	紧张	2.954	1.057	.244	2.793	.006	.872	5.035
	内疚	-.639	1.239	-.036	-.516	.606	-3.077	1.799
	恐惧	.564	1.503	.036	.375	.708	-2.395	3.522
	战战兢兢	-.223	1.225	-.016	-.182	.856	-2.635	2.188

a. 因变量：风险感知

模型		未标准化系数 B	未标准化系数 标准错误	标准化系数 Beta	t	显著性	B 的 95.0% 置信区间 下限	B 的 95.0% 置信区间 上限
1	(常量)	36.807	3.581		10.280	.000	29.749	43.864
	您的性别	2.153	2.399	.086	.898	.370	-2.575	6.881
	护理分类	-4.425	2.034	-.209	-2.175	.031	-8.434	-.415
	您现在所在省份	.282	1.382	.014	.204	.839	-2.443	3.007
2	(常量)	27.958	3.564		7.844	.000	20.931	34.984
	您的性别	-.384	2.202	-.015	-.174	.862	-4.725	3.957
	护理分类	-1.006	1.891	-.047	-.532	.595	-4.734	2.721
	您现在所在省份	.861	1.246	.042	.691	.490	-1.595	3.318
	请对您当前的情绪状态进行评估—急躁	.191	1.163	.017	.165	.869	-2.101	2.484
	哀伤	.029	1.083	.003	.027	.979	-2.105	2.163
	心烦	1.454	1.100	.139	1.321	.188	-.715	3.622
	紧张	1.253	.986	.118	1.270	.205	-.692	3.198
	内疚	-.612	1.090	-.041	-.562	.575	-2.761	1.536
	恐惧	1.457	1.200	.128	1.214	.226	-.910	3.823
	战战兢兢	2.665	.946	.231	2.817	.005	.800	4.530

a. 因变量：风险感知
