# Supplementary material for: Risk Perception and Emotion Reaction of Chinese Health Care Workers Varied During COVID-19: A Repeated Cross-Sectional Research
Source: Int J Public Health. 2021 Mar 26;66:613057. doi: 10.3389/ijph.2021.613057 (PMC8565291; doi:10.3389/ijph.2021.613057)
Supplement: Supplementary file 2 [file Image1.pdf]

Appendix A

The questionnaire for risk perception of medical staff

Thank you for participating in our investigation and we would appreciate you could answer these following questions according to your own feelings and experience. Please notice that this questionnaire should be anonymous, and all the information is strictly confidential and only allowed to use for our research purpose.

1. You often worry that there would be disputes in the medical process

〇Never ○Barely ○Sometime ○Often ○Always

2. You often worry that people who are out of control would hurt you physically

〇Never ○Barely ○Sometime ○Often ○Always

3. You often worry that high-level stress would affect your health

〇Never ○Barely ○Sometime ○Often ○Always

4. You often worry that you might be infected due to contacting with an infected person

〇Never ○Barely ○Sometime ○Often ○Always

5. You often worry that if infected you would have slim chance of being cured.

〇Never ○Barely ○Sometime ○Often ○Always

6. You often worry that you would be isolated from other people

〇Never ○Barely ○Sometime ○Often ○Always

7. You often worry about being cut or stabbed by a contaminated sharp instrument during operation

〇Never ○Barely ○Sometime ○Often ○Always

8. You often worry about being unable to detect changes in the patient's condition in time and delaying rescue

〇Never ○Barely ○Sometime ○Often ○Always

9. You often worry about how your colleagues or patients perceive you negatively

〇Never ○Barely ○Sometime ○Often ○Always

10. You often worry about making mistakes at work [单选题] *

〇Never ○Barely ○Sometime ○Often ○Always

11. You often worry that there were inadequate medical resources, hospital protection and isolation measures

〇Never ○Barely ○Sometime ○Often ○Always

12. You often worry that the situation of the epidemic would get worse [单选题] *

〇Never ○Barely ○Sometime ○Often ○Always

13. You often worry that you might have less time spending with your family

○Never ○Barely ○Sometime ○Often ○Always

14. You often worry that you might have no time to do what you want to.

○Never ○Barely ○Sometime ○Often ○Always

Appendix B

Table The Pearson correlation coefficients of negative emotions and risk perception across two periods.

	Period	N	Impatience	Sadness	Upset	Tension	Guilt	Fear	Worry
	Risk<br>1	220	0.40**	0.38**	0.41**	0.42**	0.20**	0.44**	0.44**
Perception	2	304	0.39**	0.45**	0.46**	0.48**	0.30**	0.38**	0.35**
Z			0.08	-1.06	-0.66	-0.76	-1.16	0.81	1.20

Note: ** p<0.01
